# Biophysical Scaling of Human Heart Rate: From Species to Individuals

**DOI:** 10.1016/j.jacadv.2026.102704

**Published:** 2026-03-28

**Authors:** Masato Okada, Nobuaki Tanaka, Yasushi Sakata

**Affiliations:** a Cardiovascular Center, Sakurabashi Watanabe Advanced Healthcare Hospital, Osaka, Japan; b Department of Cardiovascular Medicine, Osaka University Graduate School of Medicine, Osaka, Japan

**Keywords:** allometric scaling, heart rate, human development, ontogeny, West–Brown–Enquist framework

**What is the clinical question being addressed?**

Does the developmental decline in human resting heart rate follow the West-Brown-Enquist body-mass scaling observed across mammalian species?

**What is the main finding?**

Resting heart rate closely aligns with −1/4 allometric scaling in both sexes, suggesting biophysical scaling may extend to human development.

Resting heart rate is a central integrator of cardiovascular physiology, reflecting autonomic regulation, metabolic demand, and circulatory efficiency. Across mammalian species, heart rate reflects biological tempo and has been inversely associated with life expectancy.^1^ This tempo is partly captured by a conserved allometric relationship with body mass (*M*), with heart rate decreasing approximately as *M*^*−1/4*^.^2^ This quarter-power scaling is formalized within the West-Brown-Enquist framework and is thought to arise from fundamental constraints on metabolic energy distribution imposed by resource distribution networks.

Despite extensive evidence across species, it remains unclear whether this biophysical scaling principle also governs changes in heart rate during human development. Resting heart rate declines from infancy to adulthood, a transition traditionally attributed to cardiac growth, maturation of autonomic control, and reduced metabolic rate per unit *M*. However, whether these developmental changes quantitatively conform to the −1/4 scaling has not been examined. If this principle applies, *M* may aid interpretation beyond age-based reference ranges.

Accordingly, we hypothesized that age-dependent changes in human resting heart rate follow quarter-power scaling and tested this across the human lifespan by assessing concordance between resting heart rate and the M-based −1/4 prediction.

## Methods

We examined the relationship between resting heart rate and *M* across human development using publicly available reference data sets. Age- and sex-specific resting heart rate values were obtained from published reference tables based on the National Health and Nutrition Examination Survey (NHANES) 1999 to 2008 across predefined age groups (<1 to ≥80 years).^3^ *M* values for ages 0 to 20 years were interpolated from the World Health Organization and Centers for Disease Control and Prevention age-in-months reference curves.^4^^,^^5^ *M* for age ≥20 years was calculated from NHANES weight data. Primary analyses used median values.

Within the West-Brown-Enquist framework,^2^ whole-organism metabolic rate is expected to scale with *M* approximately as *M*^*3/4*^, implying *M*^*−1/4*^ scaling for metabolic rate per unit *M*. Physiological frequencies (eg, heart rate) are predicted to follow the same scaling. Accordingly, we denote this mass-dependent frequency as *f(M)* (∝ *M*^*−1/4*^), which reflects “physiological tempo” as the inverse of the time scale of recurrent physiological processes. Because *f(M)* is not directly observable, resting heart rate was used as its empirical proxy.

Allometric scaling was evaluated by comparing the age-related trajectory of median resting heart rate with the quarter-power–predicted trajectory based on age- and sex-specific median *M*. Sex-specific log-log linear regressions of median resting heart rate on median *M* were performed to estimate the allometric exponent β and its CIs, along with the coefficient of determination (R^2^). Sensitivity analyses included inverse-variance–weighted regression using NHANES age-group mean heart rates and SEs, a parametric bootstrap, and age-adjusted models including age as a covariate. We tested whether β differed from −0.25 using a 2-sided t test of the regression coefficient. Departures from log-log linearity were assessed by testing a quadratic term in mean-centered log(*M*) to reduce collinearity. Potential sex differences were evaluated using a sex × log(*M*) interaction term.

This study used deidentified, aggregated data; informed consent was waived, and the Institutional Review Board of Sakurabashi Watanabe Advanced Healthcare Hospital deemed the study exempt from ethics review. The data sources are available online (NHANES; /Centers for Disease Control and Prevention growth references).

## Results

### Age-related changes in resting heart rate

**Figure 1:**
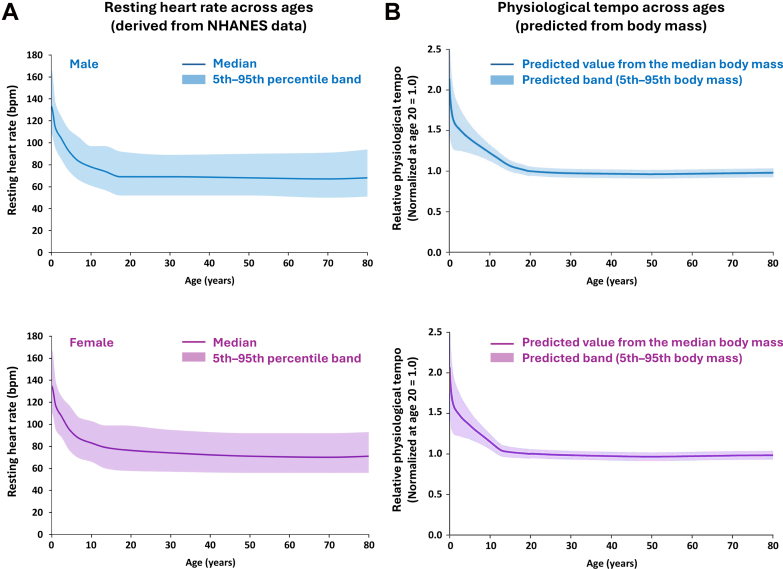
**Life-Course Scaling of Human Heart Rate and Physiological Tempo** (A) Age- and sex-specific median resting heart rate from NHANES; shaded areas indicate 5th–95th percentiles. (B) Physiological tempo predicted from body mass by quarter-power scaling, normalized to 1.0 at age 20; shaded areas indicate the predicted range derived from the 5th–95th percentiles of body mass. NHANES = National Health and Nutrition Examination Survey.

Resting heart rate declined from infancy to adulthood (Figure 1A). The median values decreased from approximately 128 to 130 beats/min in infancy to 71 to 76 beats/min in adulthood, with minimal change thereafter. This developmental pattern paralleled the age-related change in physiological tempo as predicted from *M* by quarter-power scaling (Figure 1B).

### Allometric scaling of heart rate with body mass

Using age- and sex-specific medians, the allometric exponent was close to −1/4 in both sexes (males −0.281 [95% CI −0.323 to −0.238], females −0.248 [−0.286 to −0.211]; both CIs included −0.25), with no sex × log(*M*) interaction (*P* = 0.463). The log-log relationship was highly linear (R^2^ = 0.966), and the quadratic term was not significant (*P* = 0.077 in males; *P* = 0.271 in females). Results were similar using age-group mean heart rates (weighted β: −0.242 [95% bootstrap interval −0.246 to −0.237] in males and −0.248 [−0.253 to −0.241] in females). Age-adjusted models yielded comparable estimates (β = −0.272; 95% CI: −0.332 to −0.213 in males; β = −0.236; 95% CI: −0.288 to −0.183] in females), with no sex × log(*M*) interaction (*P* = 0.344).

## Discussion

This analysis suggests that the developmental decline in human resting heart rate is consistent with the −1/4 allometric scaling predicted by the West-Brown-Enquist framework. Although this scaling law is well established across mammalian species, its applicability to human development has not been previously quantified. Using age- and sex-specific reference data, we found that resting heart rate from infancy through adulthood is well approximated by *M* using a single scaling exponent, with similar estimates in males and females. These findings suggest that resting heart rate may serve as a practical index of physiological tempo and biophysical scaling principles may extend to human cardiovascular development.

Our approach has limitations. Resting heart rate and *M* were obtained from different sources during childhood, and analyses used age-group aggregates rather than individual-level data. Therefore, the observed goodness-of-fit may overstate concordance at the individual level and may not reflect individual-level relationships. This concern is most relevant in early infancy, where interindividual variability is substantial and progressively narrows with growth. Because the West-Brown-Enquist framework assumes invariant terminal units within resource distribution networks, whether the −1/4 exponent applies throughout infancy or instead reflects population-level convergence remains uncertain.

*M*–based scaling may provide a reference for physiological tempo, but the observed 5th–95th percentile range exceeded that predicted from *M*, suggesting additional sources of interindividual variability. Broadly, population-level outcomes may likewise reflect factors beyond *M* alone. For example, women are generally smaller (implying a higher tempo) yet live longer, and *M* shows a U-shaped relationship with survival. Future studies using individual-level data should test whether *M*–adjusted heart rate metrics improve interpretation beyond age-based references.

## Conclusions

Human resting heart rate across development closely aligns with the −1/4 scaling predicted by biophysical theory.

## Funding support and author disclosures

The authors have reported that they have no relationships relevant to the contents of this paper to disclose
